# Quantized electronic transitions in electrodeposited copper indium selenide nanocrystalline homojunctions

**DOI:** 10.1038/s41598-021-83526-0

**Published:** 2021-02-17

**Authors:** Shalini Menezes, Anura P. Samantilleke, Bryon W. Larson

**Affiliations:** 1 InterPhases Solar, Moorpark, CA USA; 2 Universidade de Minho, Braga, Portugal; 3 National Renewable Energy Laboratory, Golden, CO USA

**Keywords:** Materials science, Nanoscale materials, Electronic properties and materials

## Abstract

Pairing semiconductors with electrochemical processing offers an untapped opportunity to create novel nanostructures for practical devices. Here we report the results of one such pairing: the in-situ formation of highly-doped, interface-matched, sharp nanocrystalline homojunctions (NHJs) with single step electrodeposition of two copper-indium-selenide (CISe) compounds on flexible foil. It produces a homogenous film, comprising inherently ordered, 3-dimensional interconnected network of *pn*-CISe NHJs. These CISe NHJs exhibit surprising non-linear emissions, quantized transitions, large carrier mobility, low trap-state-density, long carrier lifetime and possible up-conversion. They facilitate efficient separation of minority carriers, reduce recombination and essentially function like quantum materials. This approach mitigates the material issues and complex fabrication of incumbent nanoscale heterojunctions; it also overcomes the flexibility and scale-up challenges of conventional planar *pn* junctions. The self-stabilized CISe NHJ film can be roll-to-roll processed in ambient atmosphere, thus providing a promising platform for a range of optoelectronic technologies. This concept exemplified by CISe compounds can be adapted to create nano-scale *pn* junctions with other inorganic semiconductors.

## Introduction

Semiconductor *pn* junctions form the foundation of most optoelectronic devices. High performance planar *pn* junctions often require complex, expensive processing techniques with limited scalability. Fabricating materials systems with low-cost processes that intrinsically host nano-scale *pn* junctions, such as bulk heterojunctions (BHJs) offers an attractive alternative platform for optoelectronic technologies. Solar cell efficiencies for perovskite quantum dot, organic BHJ and polycrystalline perovskite devices have reached 18–25% range^1^. But significant challenges remain to improve their stability, toxicity, upscaling and reproducibility^2–6^. Here we show how pairing certain inorganic semiconductors, such as copper indium selenide (CISe) compounds with electrochemical processing produces high quality nanostructures that could potentially overcome such issues to enable realistic electro-optic device applications.

The CISe family of ordered defect chalcopyrites (ODC) compounds features a unique combination of desirable functional properties, including: high optical-absorption, high defect tolerance, low toxicity, exceptional longterm stability and established performance of CuInSe_2_-alloys for over 20% efficiency. The CISe ODCs offer further choice of direct bandgaps (E_g_) in the 1.04–1.34 eV range and intrinsic doping with shallow donor and acceptor defects that imparts *n*- or *p*-type conductivity^7–9^. The intrinsic doping of CISe ODCs with shallow donor (In_Cu_, V_Se_) and acceptor (V_Cu_) defects has been theoretically established^8^. The defects impart *n*-type or *p*-type conductivity to the CISe nano grains. These attributes remain largely unexploited for device fabrication. For instance, the dual-type conductivity of CISe-ODCs could realize distinct nano-scale *pn* junctions^10^. Additionally, the CISe-ODC compositions are self-stabilized and thus allow single step electrodeposition (SSE) of stoichiometric compounds from a single aqueous electrolyte, which greatly simplifies upscaling to roll-to-roll manufacturing in ambient atmosphere^11,12^. This work exploits both ODC attributes to form nanocrystalline homojunction (NHJ) films that could overcome the above issues of the state-of-the-art BHJs and similar nanostructure.

Unravelling the reaction mechanism for CISe formation enabled the SSE of stoichiometric CuInSe_2_, CuIn_3_Se_5_, or mixed CuInSe_2_-CuIn_3_Se_5_ compositions on stainless steel (SS) foil^13^. A brief low temperature air anneal stabilizes the intrinsic defect distribution and crystallizes the CISe into 4–35 nm grains. The nanocrystalline CISe films comprise strongly bound, spatially resolved, interlinked, space-filling grains, with excellent adhesion^10^. Initial electro-optical studies indicated that the SSE-made CuInSe_2_-CuIn_3_Se_5_ mixture offered surprising potential for efficient and stable NHJ devices. It revealed unexpected optoelectronic attributes related to the morphology, conductivity and composition of SSE-made CISe films. For instance, electrochemical electroreflectance (EER) spectra for the CISe film indicated high structural order and well crystallized chalcopyrite structured grains of CuInSe_2_ and CuIn_3_Se_5_; and CuInSe_2_-CuIn_3_Se_5_ (≡ CuIn_2_Se_3.5_) complexes^10^. An unexpected trend was discerned, showing lower photocurrent transients for films with CuIn_2_Se_3.5_ composition than for the films with excess non-complexed CuInSe_2_ or CuIn_3_Se_5_^13^. These first results highlight the unique and intriguing aspects of specific CISe compositions. They warrant in-depth probing into their electro-optical properties and their relationship to film composition and morphology to fill in the knowledge gaps. A profound understanding will enable exploiting the attributes of the SSE-made CISe films for efficient and easily manufactured *pn* NHJ devices. Here we combine photoluminescence (PL), time-resolved microwave conductivity (TRMC), capacitance/impedance (C/I) and atomic force microscopy (AFM) methods to uncover more remarkable aspects, concerning the local morphology and carrier dynamics for the CISe NHJ films.

## Results and discussion

**Figure 1:**
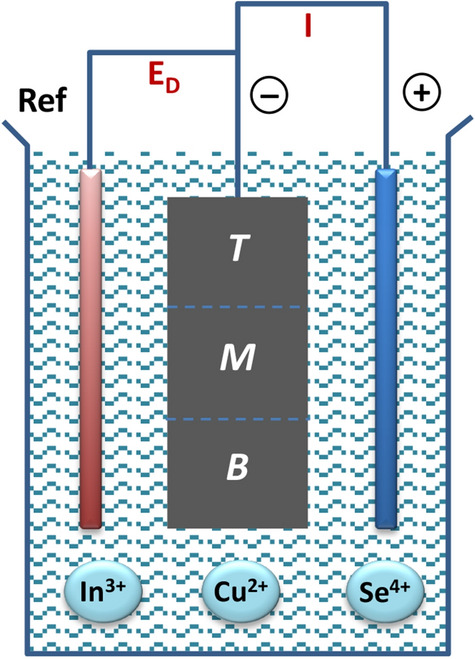
SSE of CISe from Cu^+^/In^3+^/Se^4^ electrolyte on top (*T*), middle (*M*) and bottom (*B*) sections of foil electrode.

SSE produces CISe ODC films from a Cu^2+^/In^3+^/Se^4+^ solution at a fixed potential, Fig. 1. SSE-made and briefly air-annealed CISe films were analyzed for composition and thickness with X-ray fluoresence (XRF). Table 1 lists the properties and the methods used for further characterization. Two sizes of samples were selected, based on composition, visual inspection and initial photocurrent response. The top electrical contact creates composition and thickness gradients. Films S1, S2 and S3 comprised 3 × 5 cm^2^ strips with graded composition for the (*T*) top, (*M*) middle and (*B*) bottom sections along the strip length, Fig. 1, Table 1. The area for films S3, S5, S6 was ~ 3 cm^2^.

**Table 1:** CISe film properties.

CISe film	Area	Ratio			Thickness µm	Analysis tool
		Cu	In	Se		
S1	*T*	1	1.5	3.9	1.78	PL, TRMC
	*M*	1	1.9	3.7	1.01	
	*B*	1	2.0	3.7	0.98	
S2	*T*	1	1.3	3.5	2.17	PL, TRMC
	*M*	1	1.5	3.6	1.27	
	*B*	1	2.5	3.7	1.28	
S3		1	1.0	2.0	1.95	TRMC
S4	*T*	1	1.2	2.2	1.80	TUNA
	*M*	1	1.3	2.1	1.15	
	*B*	1	1.5	1.9	0.97	
S5		1	2.4	3.4	1.80	C/I
S6		1	3.0	4.3	2.40	EBIC

**Figure 2:**
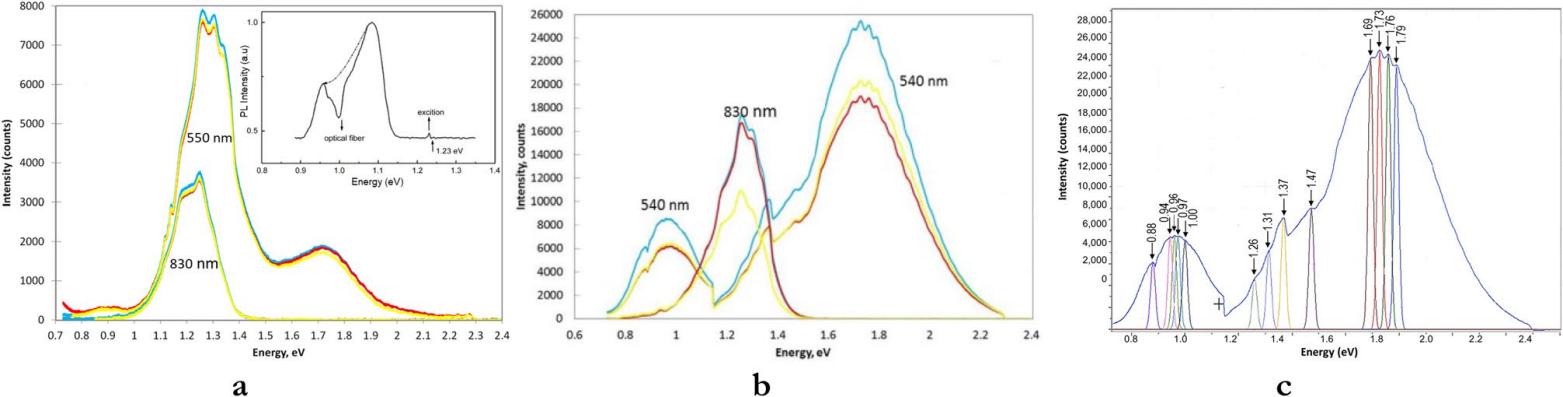
PL spectra using 532 nm laser with 830 and 540 nm filters for *T* (blue) and *M* (red) *B* (yellow) sections of (**a**) CISe S1, (inset) PL of CuIn_3_Se_5_ crystal, Ref^14^; (**b**) CISe S2; and (**c**) CLS peak analysis for CISe S2(*T*).

PL was measured with a 532 nm laser for the *T, M* and *B* sections, CISe films S1 and S2, Table 1. Figure 2a shows two sets of composite spectra for film S1 obtained with 830 and 540 nm long pass filters at 300 °K. The portion of the two PL spectra between 1.0 and 1.4 eV qualitatively corresponds to a PL spectrum for the single crystal CuIn_3_Se_5_ at 4.8°K (inset), which includes emissions from both CuInSe_2_ and CuIn_3_Se_5_^14^, but without the superimposed fine structure that is evident for the new sets of spectra. The 830 nm filter blocks emissions from energies > 1.4 eV and selectively transmits the PL from the shallow states near the edge of E_g_ for CuIn_3_Se_5_, Fig. 2a. The expanded range spectra obtained with the 540 long pass filter show additional features, including a low bump for CuInSe_2_ (0.95 eV), large peak (1.3 eV) for higher order ODCs and a smaller peak (1.73 eV). The 3 sections of film S1 essentially show the same PL intensity and spectral features in both sets of spectra, which likely reflects the nonexistence of significant composition gradient for film S1.

Figure 2b also compares 2 sets of PL spectra for the *T, M, B* sections but with varying compositions for film S2, Table 1. The PL spectrum discontinuity seen at ca. 1.15 eV arises from changing detectors. The PL spectra are qualitatively similar to those for S1 but PL intensity for analogous peaks is much higher. Here, the relative PL intensity varies with the compositions for S2(*T*), S2(*M*), S2(*B*), sections while the spectral features remain the same. With the 830 nm filter, S2(*T*) and S2(*M*), having Cu:In:Se ratio closer to CuInSe_2_ show similar PL intensity, while the In-rich S2(*B*) with composition closer to CuIn_2_Se_3.5_ exhibits lower PL response. With the 540 nm filter, S2(*T*) still shows higher intensity but the peak PL intensity of the S2(*M*) slightly lower than for S2(*B*). The presence of varied bulk content of these different stoichiometric ratios is consistent with a respectively weighted intensity of those two regions of PL. The trend for reversal of PL peak intensity could have resulted from local inhomogeneity wherein the complexed and non-complexed [CuIn_3_Se_5_] or [CuInSe_2_] grains respond somewhat differently to the light transmitted by 540 and 830 filters. The intensity variation trend for S2(*B*) or S2(*M*) with different filters may not be significant, the overall PL emission intensity could be associated with the extent of the [CuInSe_2_–CuIn_3_Se_5_] complexation in the film as previously observed with other techniques^13^. The previously trend of lower photocurrent transients was seen for CISe films comprising CuInSe_2_-CuIn_3_Se_5_ complex (≡ CuIn_2_Se_3.5_). Conversely, films comprising excess [CuIn_3_Se_5_] or [CuInSe_2_] show higher PL intensity (and faster charge transport) than the films comprising [CuIn_3_Se_5_] ≈ [CuInSe_2_].

Surprisingly, the overall PL spectral features and the superimposed sub-E_g_ fine structure for the 3 regions of film S2 appear identical despite significant composition variation that merely reduces the PL emission intensity from S2(*T*) to S2(*B*)*,* Fig. 2b. By comparison to Fig. 2a, the Fig. 2b spectra show: high CuInSe_2_ PL peak; enhanced PL for CuIn_3_Se_5_; similar PL feature at 1.4 eV; much larger PL at 1.73 eV; 3.5 times higher PL intensity; and more intricate fine structure. The fine structure likely arises from specific sub-E_g_ emissions, originating from shallow native defect states near the conduction band (CB) or valence band (VB) edge of the nanostructured S1 and S2 films. They are located near the E_g_s of CuInSe_2,_ CuIn_3_Se_5_ and CuIn_5_Se_8_ and an unidentified feature at 1.73 eV. The predominantly near-E_g_ emissions correspond to free-carrier-to-shallow defect transitions and band-to-band transitions from specific states. For example, the transition peaks at: 1.20 eV ≡ shallow donor state in CuIn_3_Se_5_; 1.26 eV ≡ CB; 1.31 eV and 1.37 eV ≡ energy levels inside CB or ≡ shallow donor states from CuIn_5_Se_8_. Such shallow defect transitions enable efficient charge separation within the *p-*CuInSe_2_/*n-*CuIn_3_Se_5_ complex, as evidenced by the EER data^10^.

The PL spectrum in Fig. 2c uses classical least squares calibration to identify discrete states and components of S2 that emit at specific wavelengths. The energy of each fine-peak corresponds to emitted light and thus determines the energy of the defect level. The discretely spaced, quantized energy states correspond to known shallow defect transition. PL emissions between 0.8 and 1.2 includes: peaks within the CuInSe_2_ E_g_, such as: 1.00 eV ≡ (CB → V_Cu_) or ≡ (In_Cu_ → VB); 0.93 eV ≡ (In_Cu_ → V_Cu_); 0.88 eV ≡ (V_Se_ → V_Cu_) or (In_Cu_ → VB). The PL emissions from 1.2 − 1.5 eV occur near the E_g_s (1.23, 1.34 eV) of CuIn_3_Se_5_ and CuIn_5_Se_8_. Interestingly, the CLS fitting shows that the superimposed fine structure is typically spaced at 30 mV. It is intriguing that the composition variation does not change the fine structure.

The PL data implies that the optical and electronic properties of the CISe film are intimately related via a quantum system, comprising quantized energy levels. The unexpected broadening in PL spectral range resulting from the above-E_g_ emission peak, centered around 1.73 eV is seen for both samples S1 and S2, but is more pronounced for film S2. It appears to encompass the E_g_s of In_2_Se_3_ (1.8 eV) and Se (1.7 eV). The CISe NHJ band structure could contain defect states associated with these compounds, which were incidentally not discerned by other methods^10–13^. Alternatively, the blue shift may ensue from grain size effects. Intra-grain and band-to-band transitions were detected by EER for the *p-*CuInSe_2_/*n-*CuIn_3_Se_5_ complex^10^. The peak also shows ~ 30 mV spaced fine structure, e.g. 1.69, 1.73, 1.76, 1.79 eV. The apparent quantization of states likely results in quantum confined nano-regions in the CISe film that possibly contribute to this large PL emission. The emission of multiple and narrow emission lines is a hallmark of photon upconversion in nanoparticles. It entails stepwise low energy photon absorption, energy transfer and the emission of high energy photons, i.e., quantized electronic transistions can transmute sub-E_g_ photons into higher energy (above-E_g_) photons. Such upconversion phenomena are often observed in core–shell nanoparticles and transition metals^15–18^. In the present scenario, it apparently results from the near quantum dot sized CISe nanocrystals that fortuitously lead to the above-E_g_ quantized states. Significantly this approach allows quantum confinement without introducing external rare earth dopants into the host material as in state-of-the-art upconversion^18^. Linking this self induced nonlinear optics phenomenon to the CISe NHJ electronic structure could reveal a new avenue for electro-optical applications.

Transient photoconductivity analysis was carried out using TRMC for same films as PL, S1 and S2 along with a third stoichiometric CuInSe_2_ film, S3. The In-rich sections, S1(*B*) and S2(*B*) were chosen for these measurements, Table 1. TRMC measures the changes in continuous probe ~ 9 GHz microwave power induced by an optical pulse excitation of a free-carrier generating sample within a high sensitivity resonant cavity^19,20^. The CISe films: S1(*B*), S2(*B*), and S3, were delaminated from the SS foils and transfered to a low-dielectric loss quartz substrate using a double-sided tape transfer method. The samples were excited by 10 Hz 500 nm laser pulses during continuous dry N_2_ purge.

**Figure 3:**
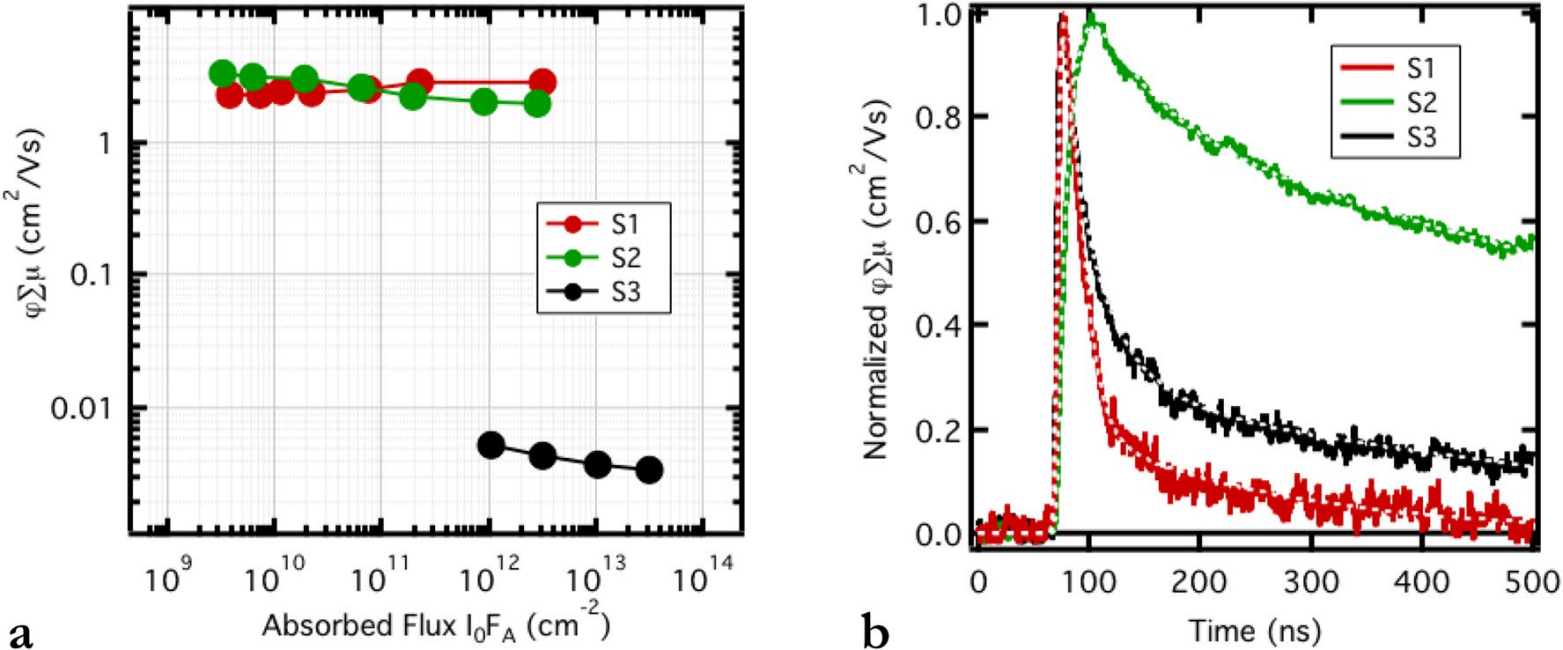
Plots of (**a**) φΣμ versus absorbed photon flux and, (**b**) normalized photoconductivity transients for CISe films S1(*B*), S2(*B*) and S3 with fits shown as dashed gray lines.

Figure 3 shows the TRMC results for the samples S1(*B*), S2(*B*), and S3. The TRMC figure of merit is given by φΣμ, corresponding to the peak product of free-carrier generation yield (φ) and sum of free electron and hole mobilities (Σμ). The φΣμ was quite low at (3.9 ± 0.5) × 10^–3^ cm^2^/Vs for sample S3, with CuInSe_2_ composition. Bi-exponential fits of the 500 ns photoconductivity transient decays gave an average lifetime approaching the instrument response of the microwave cavity of less than 30 ns. Sample S1(*B*) with ~ CuIn_2_Se_3.5_ composition showed dramatically improved φΣμ of 1.9 ± 0.4 cm^2^/Vs, nearly 1000 × larger than S3, yet the average weighted lifetime extracted from the fitted photoconductivity transient was still as short as S3 and nearly instrument response limited; TRMC free carrier lifetimes this short are not promising for useful device applications that rely on bulk charge transport. In contrast, sample S2 (*B*) with ~ CuIn_3_Se_5_ stoichiometry exhibited a φΣμ of ca. 3 cm^2^/Vs with a remarkable average weighted photoconductivity lifetime of 740 ns (fitted from 500 ns of data). To verify the long lifetime in this sample, the signal collection window was expanded to 2 µs and confirmed the lifetime at 727 ns. Also in contrast to S3 and S1(*B*) samples, the S2(*B*) exhibited a strong dependence of photoconductivity lifetime on excitation intensity; above an absorbed photon flux of 1 × 10^10^ cm^−2^ the lifetime decreased, but below this absorbed flux there was no intensity dependence. One explanation may be that the sharp *pn* NHJs in this composition serve as charge recombination centers at higher carrier densities, especially given that for nanocrystals of this size, the microwave photoconductivity signal is dominated by inter-grain carrier dynamics. Even so, the TRMC data confers with the PL data for S1 and S2. It implies that the observed shallow defect transitions may  be responsible for the extraordinary attributes of film S2, and likely contribute to its long carrier lifetime.

**Figure 4:**
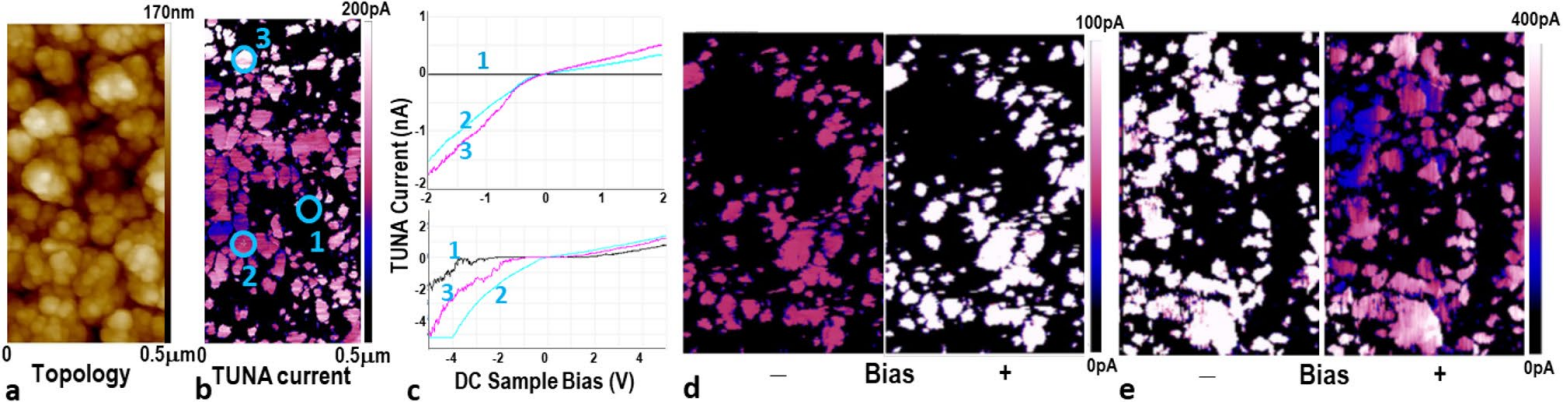
Micrographs of (**a**) AFM topology and (**b**) TUNA current with DC bias: 1 V in main scan, − 1 V in dark lift scan, mapping on same 0.5 µm^2^ area of film S4; (**c**) I–V curves 1, 2, 3 obtained at spots 1, 2, 3 on AFM micrograph (**b**), for two ranges of negative and positive bias; vertical color scalebars ≡ photocurrent range. TUNA photocurrent mapping under positive and negative bias for (**d**) *p-*grains in S4(*T)* area and (**e**) *n*-grains in S4(*B*) area; AC bias: 5 V in lift height: 100 nm.

The following electrical characterization provides further insights into the electronic properties and structure of SSE-made CISe films. AFM topography in Fig. 4a confirms the nanocrystalline grain structure for CISe film S4. Tunneling AFM (TUNA) map of Fig. 4b on the same area of film S4 discerns substantial current variation for the grains, which range from highly conducting to semi-insulating, as denoted by the tip-current range bar (0–200 pA). Comparing the micrographs of Fig. 4a, b reveals that the large agglomerates comprise smaller grains with different conductivities. Figure 4c shows I-V curves from individual domains, denoted by circles on Fig. 4b micrograph. The scans between − 1.5 to 1.5 V show high TUNA currents from the brighter domains 2 and 3. Domain 1 (dark spot) was not conductive in this range, but became more conductive under higher bias, in the − 5 V to 5 V range (lower curves of Fig. 4c). A much steeper I-V curve with negative bias than positive bias is consistent with the photocurrent mapping for this region, confirming higher conductivity for domain 1 within the film.

Shining a laser on a *pn* junction generates electron–hole pairs that can significantly affect the minority carrier concentration, hence the Fermi energy level and the width of the depletion region. This enables the TUNA photocurrent to distinguish the predominant conductivity type. Figure 4d, e demonstrate the changes in photocurrent polarity with applied bias for the S4(*T*) and S4(*B*) areas, Table 1. The images show that the *p*-CISe grains (4d) generate higher photocurrent under positive bias, whereas the *n*-CISe grains (4e) generate more photocurrent under negative bias. The switching of photocurrent polarity with applied bias provides visual evidence that the currents originate from individual *p-*CISe and *n*-CISe domains, coexisting within the same film. Both types of grains were present in the entire film, although the S4(*T*) and S4(*B*) areas exhibited stronger *p*-type and *n*-type response, respectively. Note that the different photocurrent scale bars in Fig. 4d, e correspond to different doping density (N_D_) ranges in *p*- and *n-*grains. These observations are consistent with the Cu-rich and In-rich compositions for the S4(*T*) and S4(*B*) sections in Table 1.

**Figure 5:**
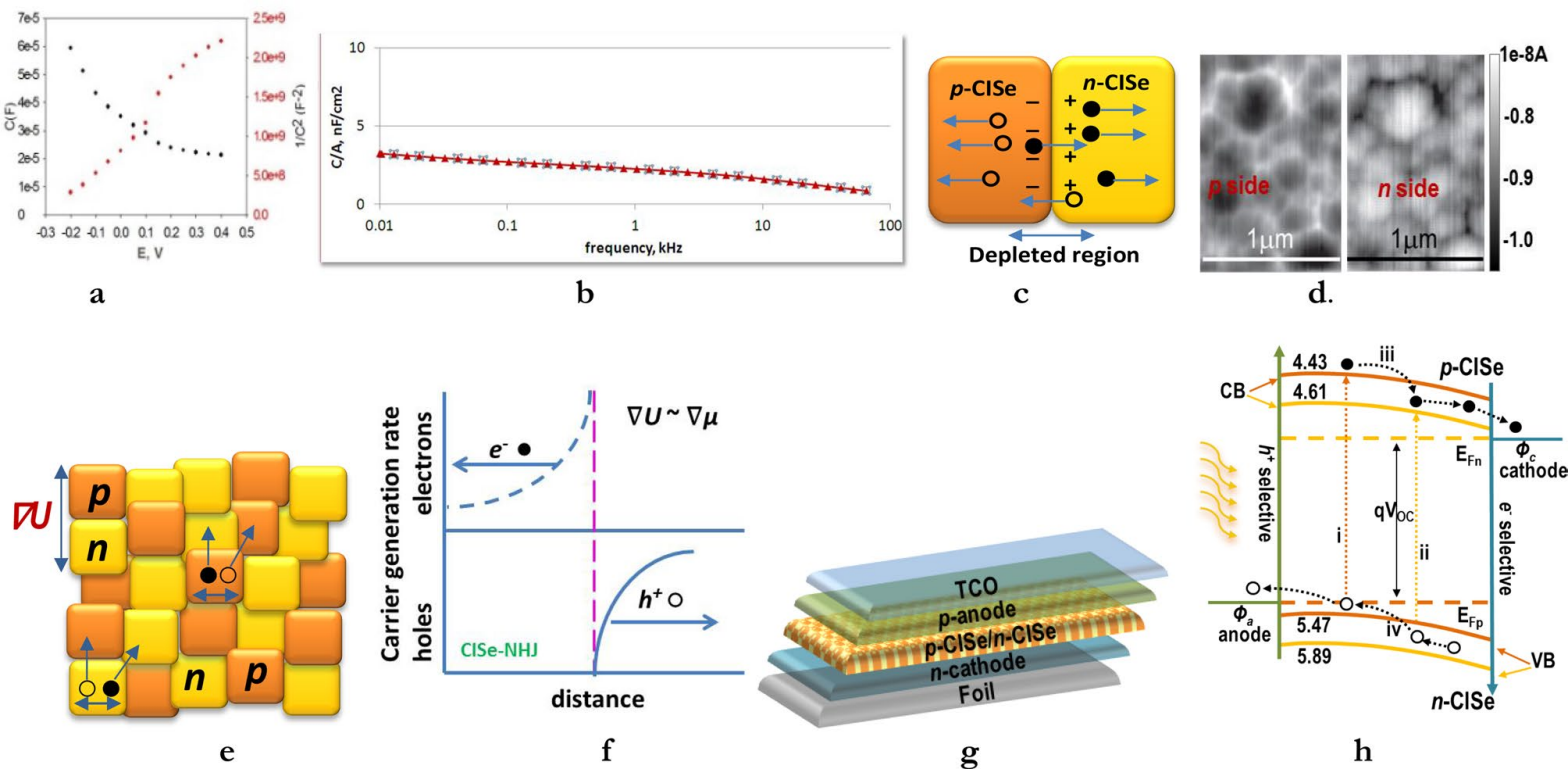
(**a**) Mott–Schottky plots of **V** versus **C** and 1/**C**^2^ and (**b**) admittance spectroscopy at 300°K for CISe/SO_3_^2^ junction with film S5; (**c**) Schematic of depleted *p-*CISe/*n*-CISe junction; (**d**) EBIC mapping on either side of SS/CISe(S6)/ITO device at 10 V; (**e**) Schematic of electron–hole pair generation and separation in *pn* NHJ; (**f**) Potential gradients ∇U ≈ ∇µ that drive device operation; (**g**) typical device structure; (**h**) band-to-band and intraband transitions in CISe NHJ.

Despite the strong photocurrent response, scanning capacitance microscopy (SCM) contrast was not detected for film S4, possibly because its N_D_ value was outside the sensitivity range, i.e. < 10^14^ or > 10^21^. Electrolyte impedance spectroscopy was performed in the frequency range of 100 kHz to 1 Hz for analogous CISe film S5. Figure 5a shows Mott–Schottky plots of bias (**V**) versus capacitance (**C**) and 1/**C**^2^ for film S5. It shows high capacitance (tens of microns), N_D_ > 10^21^ cm^−3^, and flat band potential of − 0.2 V vs. Ag/AgCl. The high values for N_D_ are consistent with the V_Cu_ and In_Cu_ defect-doped, In-rich *n*-CISe ODC such as CuIn_3_Se_5_. The *p*-CISe was close to CuInSe_2_ stoichiometry^7,10^. The widely different N_D_s, outside the SCM range for the two conductivity types is probably the reason for lack of SCM contrast. Based on this rationale and the respective defect structure, we tentatively assign the very high doping N_D_ (> 10^21^ cm^−3^) for the *n*-CISe grains and the very low doping N_D_ (< 10^14^ cm^−3^) for the *p*-CISe grains. Admittance spectroscopy results indicate minimal frequency dependence of defects even at 300°K, Fig. 5b. Frequency independence implies low density of trapping defects^21^. The Mott–Schottky and impedance data, stating the N_D_ and the absence of deep defects corroborates with theoretical treatments that the intrinsic defects in *n*-CISe are shallow^9–11^. The shallow intrinsic defects are uniformly distributed within the nano grains and most of these grains are uniformly and intrinsically doped (10^21^/cm^−3^ ≡ one doping site per nm^3^). The shallow defects allow free carrier flow without trapping, avoid degeneracy and enhance grain-to-grain current flow.

Comparing the topography and AFM micrographs for film S4 on the same 1 µm wide area indicates that the very prominent, bright nanograin agglomerates in Fig. 4a correspond to the dark, low current spots in Fig. 4b. This implies that these agglomerates comprise depleted *pn* junctions with neutralized electrical charges. Figure 5c schematic illustrates that depleted grains/junctions would occur when carrier diffusion length > grain size. This would create sharp junctions that serve as recombination barriers. In fact, surface EBIC mapping obtained from either side of a simple SS/CISe/ITO device fabricated from film S6 confirms that the grain boundaries are indeed *pn* junctions, Fig. 5d. The EBIC signal was strongest in the spaces between the larger grains. Reversing the connections switches the polarity of the EBIC contrast, verifying that signal response came from either side of the device. The sharp boundaries between light and dark areas indicate abrupt interfaces between the depleted *p*-grain and *n-*grain, forming high quality *pn* NHJ. The sharp *pn* interface acts as a barrier to recombination of minority carriers; it could enhance current flow through devices, such as solar cells. Besides illustrating the different conductivities and *pn* junction formation, the combined AFM and EBIC results map out the conductive nature of the distributed grains in addition to the physical distribution of grains.

These results indicate that under specific conditions, SSE spontaneously creates highly-ordered *p-*CISe and *n*-CISe nanocrystals that interconnect in a 3-dimensional (3*D*) network. The electrical potential gradient, ∇*U* at the *pn* NHJ interface separates the electron–hole pairs that are generated in each nanograin, Fig. 5e. The short distance to the nano *pn* junctions enables fast separation of electron and hole carriers. Electrons move through the *n*-CISe and holes through the *p*-CISe. The continuous generation of separated electrons and holes contributes to the high carrier density. This creates and maintains a chemical potential gradient, ∇μ to drive the transport of electrons and holes separately along the *p-*CISe and *n*-CISe grains, Fig. 5f. Note that standard bilayer planar *pn* junctions primarily utilize the ∇*U* while exitonic devices utilize the ∇μ^22^. Unlike either of such device configurations, the CISe NHJ has the potential to effectively utilize both of the fundamental driving forces, i.e. ∇*U * ≈ ∇*μ*, as shown in Fig. 4b. When placed between *p* and *n* electrodes the CISe NHJ layer forms a device with the configuration of: foil/*n*-cathode/*n*-CISe/*p*-CISe/*p*-anode, Fig. 5g. The band diagram in Fig. 5h shows the alignment between the NHJ device components including the quasi Fermi levels, E_F*n*_, E_F*p*_ the valence band (VB) and conduction band (CB) edges for the *n-*CISe and *p*-CISe, respectively. The band-to-band transitions in (i) *p*-CISe and (ii) *n*-CISe nanograins as well as the intra-grain (i + iii), (ii + iv) transitions nanograins were previously derived from EER spectra^10^. This combination of intra-grain and band-to-band transitions within the CuInSe_2_-CuIn_3_Se_5_ complex could efficiently separate minority carriers, moving the holes to anode and the electrons to cathode. Suitable carrier selective contacts electrodes are needed to produce highly efficient *p*-CISe/*n-*CISe NHJ devices.

Although solar cell efficiency is not the primary focus of this paper, we have tested several preliminary devices with the architecture of SS/CISe/TCO without appropriate carrier-selective electrodes; they exhibit V_OC_ of 0.6 V and photocurrent of up to 23 mA/cm^2^ under 33 mW/cm^2^ illumination. But the efficiency obtained was ~ 5%, mainly due to the low fill factor that is expected for devices without carrier selective interlayers. Yet, based on the premise of low recombination in CISe NHJ space charge region, the low fill factor likely arises from recombination at the CISe/back contact or the series resistance of the device due to poor CISe/front contact interfaces. Thus, carrier selective electrodes could essentially prevent such interface leakages to maximize device efficiency. These preliminary photovoltaic metrics are especially promising given the completely unoptimized nature of the proof-of-concept devices and the low E_g_ of the CISe absorber. Thus, if optimized CISe NHJ device can be achieved with fill factor in the range of current peroskite devices of ~ 80%^1^ the efficiency could reasonably approach the 20% efficiency benchmark.

The electronic properties concur and strongly support the PL and TRMC data. The shallow defects, high N_D_ and sharp *pn* junctions that indicate efficient charge carrier dynamics suggest that the CISe nanocrystals may work as quantum particles. Quantum confinement within CISe nanocrystals evidently generates the unusual opto-electronic properties. Such confinement could produce discrete spaced transitions within quantized states and contribute to the unexpected above-E_g_ PL emission. The fact that PL spectra are well-resolved even at 300°K attests to the unique quality of SSE-created CISe films. Only very pure and high quality crystals or artificially created quantum dot structures have shown similar transitions and non-linear absorption, and in those cases only under cryogenic conditions unlike here, where it is observed at room temperature. Moreover, the electronic nanostructure of CISe NHJ can tolerate wide composition variation, i.e. CISe films with In/Cu ratios from 1.3 to 2.5 give essentially the same PL spectra. This is in stark contrast to the precise composition and electronic configuration required for standard devices and the associated high fabrication costs. The direct deposition of CISe *pn* NHJ resolves many issues with the existing approaches, used to fabricate conventional planar *pn* junctions, perovskite/organic/inorganic bulk heterojunctions or inorganic ordered heterojunctions. Significantly, the CISe grains have highly desirable matching interfaces since they grow into each other as *pn* junctions. This finding solves a major hurdle for creating inorganic *pn* junctions. The exceptionally stable, non-toxic CISe NHJ avoids other issues of the nano *pn* junction devices, in particular the doping difficulties, separate synthesis and physical mixing of two material types. However, many of the carrier selective electrodes deployed in such devices can be utilized to produce the CISe NHJ device of Fig. 5g.

## Conclusions

Coupling electrodeposition with CISe semiconductors creates scalable thin-film of nano *pn* NHJ. Its unexpected properties, including PL spectral range broadening, quantized emissions and the long lifetime, imply non-linear behavior similar to that seen in artificially designed quantum dot structures. Quantum confinement within CISe nanocrystals evidently generate discretely spaced quantized states. Specific charge carrier transitions within these states may be responsible for their unusual opto-electronic properties. Notably, the quantum confinement is enabled without external rare earth dopants into the host material. The extraordinary attributes of specific CISe films may be linked to the reaction kinetics during SSE that create special CISe morphologies with CuInSe_2_-CuIn_3_Se_5_ complexes.

The SSE-made films comprise *n*-CISe and *p*-CISe grains co-existing in same film. The cell geometry during SSE determines the predominance of Cu-rich *p*-CISe or In-rich *n*-CISe grains in the film. The *n*-CISe exhibits much higher N_D_ and photocurrent than *p*-CISe. Shallow defects that intrinsically dope the CISe films appear to be uniformly distributed within the nano grains at the rate of one doping site per nm^3^. SSE naturally creates *3D* network of highly-doped, interface-matched, sharp, abrupt *p-*CISe/*n*-CISe NHJs in-situ. These CISe *pn* NHJ potentially utilize both of the fundamental driving forces, i.e. ∇*U * ≈ ∇*μ*, to reduce recombination, enhance carrier separation and transport, and thus boost future device performance. Band-aligned, carrier selective electrodes are sought to transition the CISe NHJ into high performance devices.

SSE presents a generic, very low-cost processing platform to create high quality *pn* NHJs that could essentially perform like crystalline 2*D* planar *pn* junctions or artificially ordered 3*D* nanostructures^1^ without their fabrication complexities. The results also call attention to the rarely explored ODC semiconductors that offer a plethora of relevant properties to produce nanostructures of technological interest. The resulting absorbers/emitters can be directly used in electro-optic devices with minimum manipulation. They offer new perspectives for processing devices with atmospheric roll-to-roll SSE. Such fabrication can impact a variety of the flexible electronics for next-generation optical communications, sensing, and imaging applications^23–27^. The resulting device roll can be fashioned into inexpensive flexible solar panels, or directly incorporated in solid-state lighting devices, photoelectrodes and other energy conversion. Overall, the SSE method efficiently balances the critical parameters for inorganic electro-optic devices: performance, stability, cost and scalability, for a variety of flexible devices in simple thin-film form factor.

## Materials and methods

The SSE of CISe films was performed in 0.15 M NaCl aqueous electrolyte containing specific Cu:In:Se concentration ratio at 60 °C. The films were deposited on 117 cm^2^ SS foil using Technic’s high speed flow cell or on a 3 cm^2^ SS foil in a beaker. Experimental conditions were varied to obtain samples with different CISe composition, including external dopants. The electrolyte pH was adjusted to ~ 2 during the deposition, with NaOH or HCl solution. Sufficient CuCl solution was added to the electrolyte to deposit roughly CuIn_2_Se_3.5_ compositions. Se was added to maintain a [Se^2^]/[Cu^+^] ratio between 3 and 6. The formation of In-rich CISe-ODC compounds requires excess of In^3+^ concentration and slightly higher pH. The films were annealed using an IR lamp (650 W) at 90% power with 2–3 s pulses, repeated 10–15 times in ambient atmosphere. Film composition and thickness were analyzed at multiple spots across the sample area with Spectro Midex XRF spectrometer.

Electrochemical characterization was performed on masked CISe samples, exposing an area of 0.25 cm^2^ to the Na_2_SO_3_ (pH 3) electrolyte held in a 3-electrode cell. Lock-in ampliflier was used in a frequency range of 100 kHz to 1 Hz to obtain capacitance values and Electrolyte Impedance Spectroscopy.

PL emission spectra were obtained at 300°K (room temperature), using Horiba’s Micros setup with a 532 nm laser excitation. Long pass filters were used to block the short wave light below 540 nm or 830 nm; the data were not corrected for the grating spectra. Contactless probing with TRMC provided relevant photoelectric response of CISe films. The technique and its application to photoresponsive materials has been described in explicit detail^19,28–34^. Since the CISe films were deposited onto SS foil, which would short the microwave cavity, the films were transferred to low-dielectric loss quartz substrates (25 × 11 × 1  mm^3^) using a delamination-and-tape-transfer process. Single wavelength excitation pulses at 10 Hz (ca. 5 ns pulse width) and 500 nm were generated by a 355 nm pumped optical parametric oscillator (Continuum) on the signal arm of the output. A series of absorptive neutral density filters were used to attenuate the excitation power over 3 orders of magnitude, and the beam area was diffused to uniformly illuminate the entire cross-section of the q-band waveguide, providing uniform optical pumping over the entire area of the sample (~ 2.5 cm^2^). The microwave cavity was purged continuously with dry nitrogen during all measurements.

AFM-TUNA measurements used bias polarity effects to distinguish between *p*-type and *n*-type grains in the film. CISe sample S1 was cut and mounted on a metal disk with Ag-paste. Topological and nano-electrical AFM-TUNA micrographs were obtained on 1 µm area, using Bruker Dimensional Icon and SCM-PIT or SCM-PTSI probes, under AC bias of 5 V, lift height of 100 nm and scan rate of 0.5 Hz. This system included Kelvin probe force microscopy (KPFM) and scanning capacitance microscopy (SCM). The dark lift mode was used to eliminate the laser effects on the minority carrier concentration, hence the Fermi energy level and the width of the depletion region. The adjunct SCM, KPFM techniques were used to discern surface variations in carrier concentration and work function between the tip/CISe sample.

EBIC micrographs were obtained by Ephemeron, Inc using Mighty EBIC 2.0 on ITO/CISe/SS device at 10 V made from film S6.
